# Supplementary material for: Heritability maps of human face morphology through large-scale automated three-dimensional phenotyping
Source: Sci Rep. 2017 Apr 19;7:45885. doi: 10.1038/srep45885 (PMC5395823; doi:10.1038/srep45885)
Supplement: Supplementary Information [file srep45885-s1.pdf]

# Heritability maps of human face morphology through large-scale automated three-dimensional phenotyping

**Dimosthenis Tsagkrasoulis**[1], **Pirro Hysi**[2], **Tim Spector**[2], **and Giovanni Montana**[1,3,*]

[1]Department of Mathematics, Imperial College London, SW7 2AZ, London, UK
[2]Department of Twin Research and Genetic Epidemiology, King's College London, SE1 7EH, London, UK
[3]Department of Biomedical Engineering, King's College London, SE1 7EH, London, UK
[*]giovanni.montana@kcl.ac.uk

## ABSTRACT

The human face is a complex trait under strong genetic control, as evidenced by the striking visual similarity between twins. Nevertheless, heritability estimates of facial traits have often been surprisingly low or difficult to replicate. Furthermore, the construction of facial phenotypes that correspond to naturally perceived facial features remains largely a mystery. We present here a large-scale heritability study of face geometry that aims to address these issues. High-resolution, three-dimensional facial models have been acquired on a cohort of 952 twins recruited from the TwinsUK registry, and processed through a novel landmarking workflow, GESSA (Geodesic Ensemble Surface Sampling Algorithm). The algorithm places thousands of landmarks throughout the facial surface and automatically establishes point-wise correspondence across faces. These landmarks enabled us to intuitively characterize facial geometry at a fine level of detail through curvature measurements, yielding accurate heritability maps of the human face (www.heritabilitymaps.info).

## Supplementary Information

### 0.1 Supplementary Figures

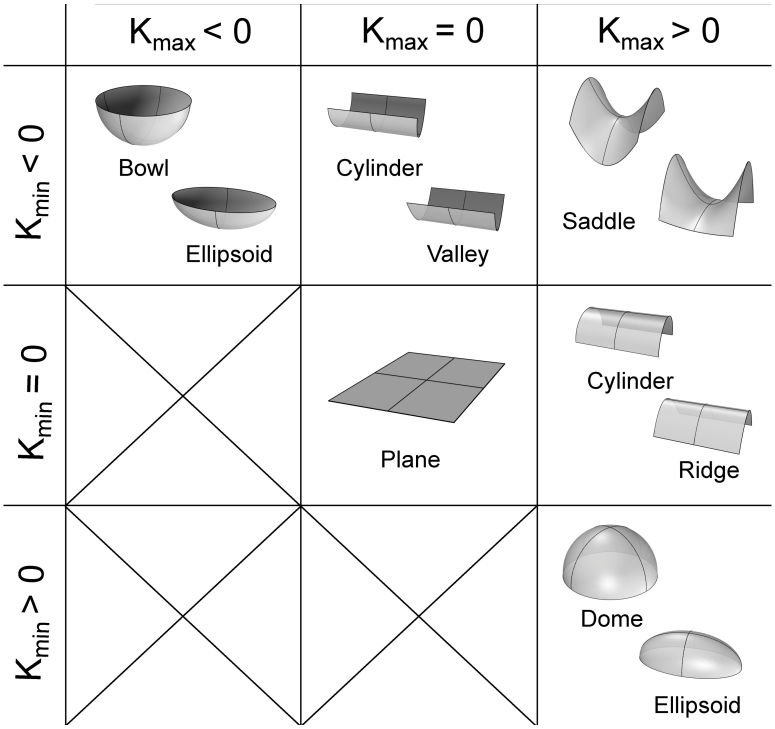


**Figure 1. Principal curvatures and shape characterization.** General classification of shapes based on the signs of the two principal curvatures. While principal curvatures include all information about the curvature at a point, both numbers are needed in order to get meaningful shape categorizations.

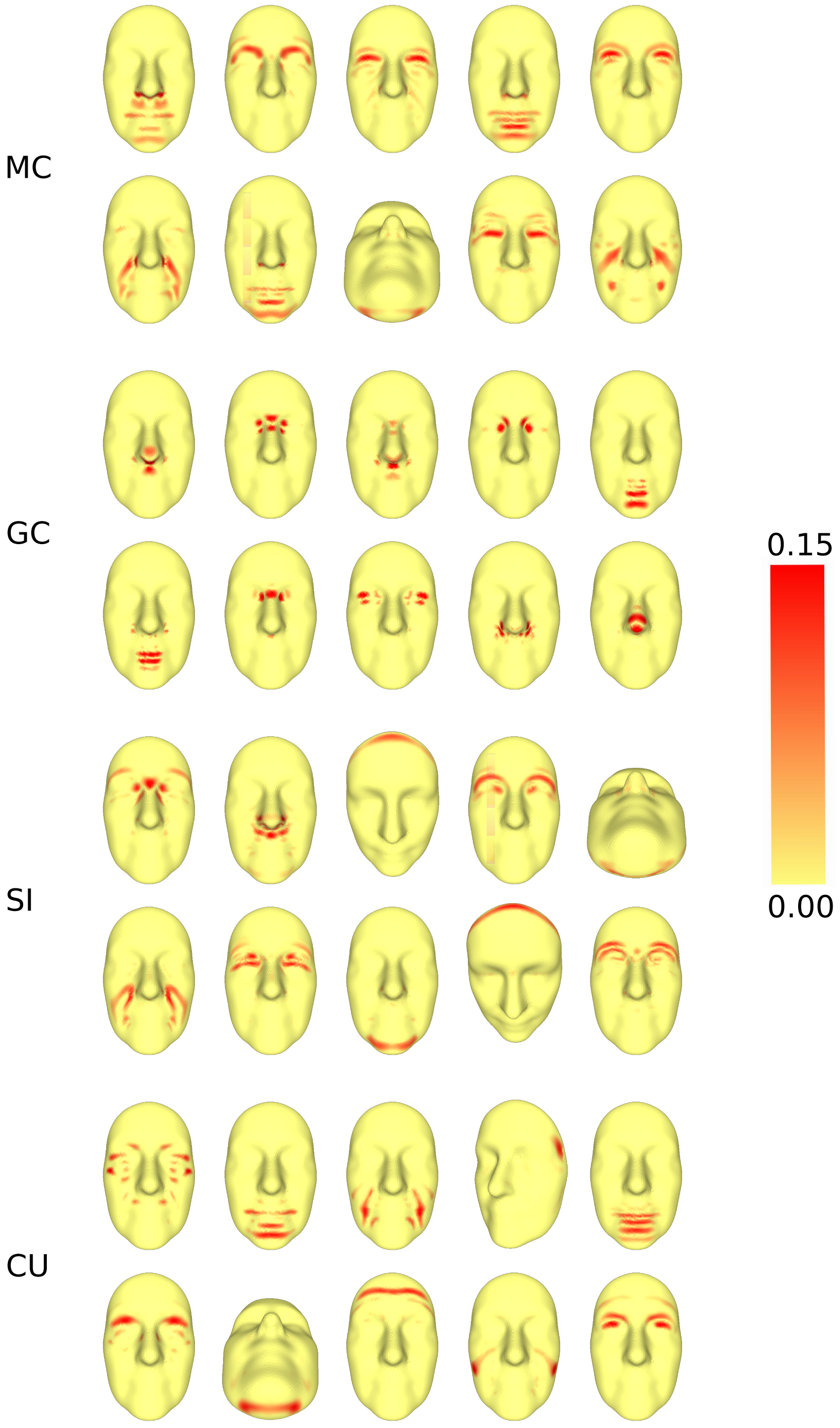


**Figure 2. Eigenfaces associated to the largest principal components for each curvature index.** For each curvature index, the faces are arranged in decreasing order, from left to right.

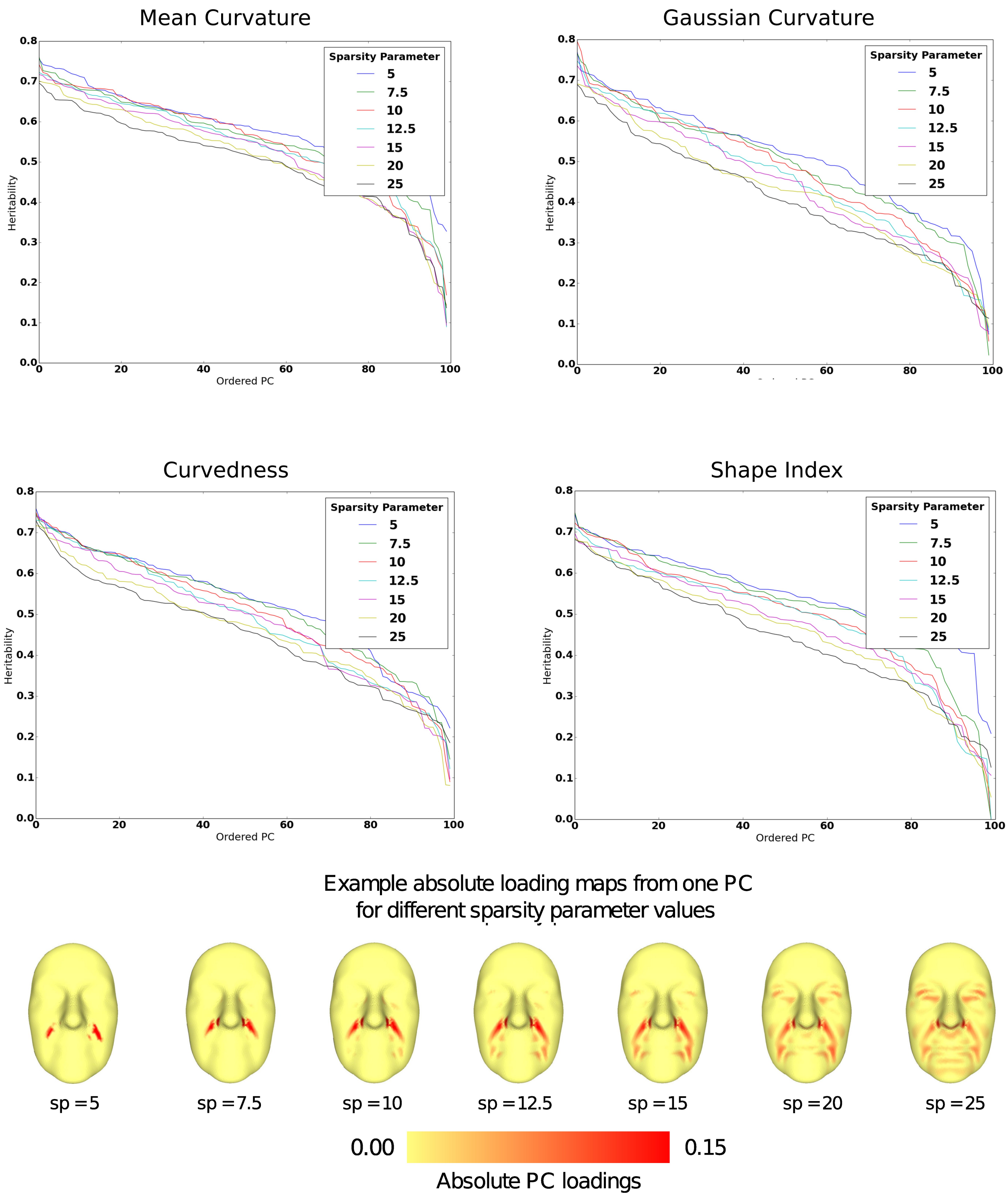


**Figure 3. Effect of sparsity parameter on composite curvature trait heritability analysis.** Different sparsity parameters were tested in order to assess the parameter's contribution on the heritability study. Each plot shows the sorted heritability values for the first 100 sPCs per curvature descriptor and for 7 different sparsity parameters. Heritability estimates showed similar behavior irrespective of how sparse the components were. The facial maps depict the absolute loadings of an example composite trait (Mean curvature descriptor) for different sparsity parameter values and their computed heritability values.

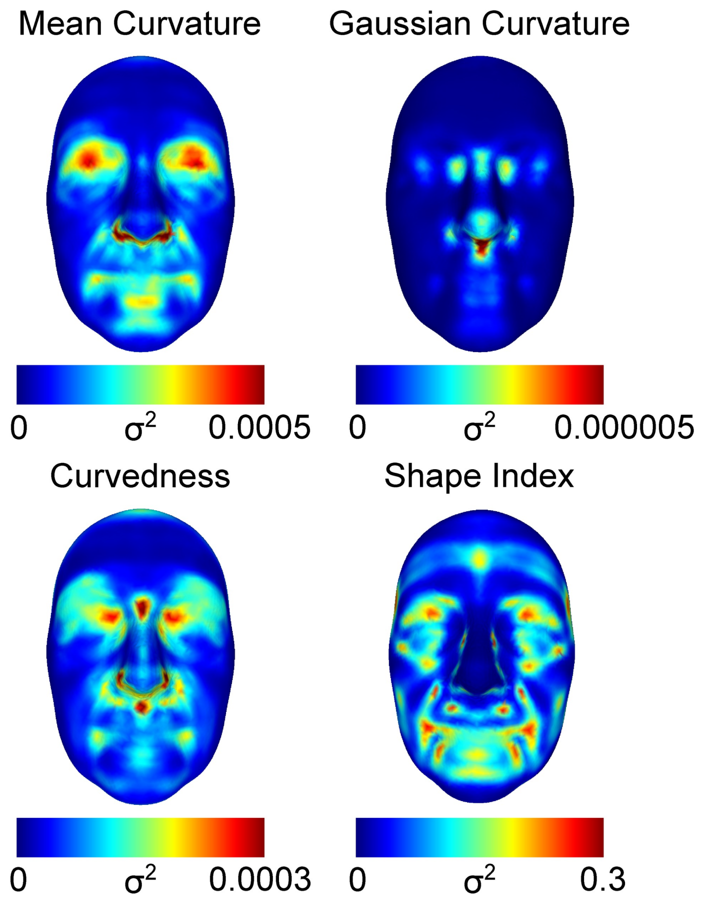


**Figure 4. Curvature variance Maps.** The maps illustrate the variance of curvature values on all landmarks, computed from the full dataset of 952 TwinsUK subjects.

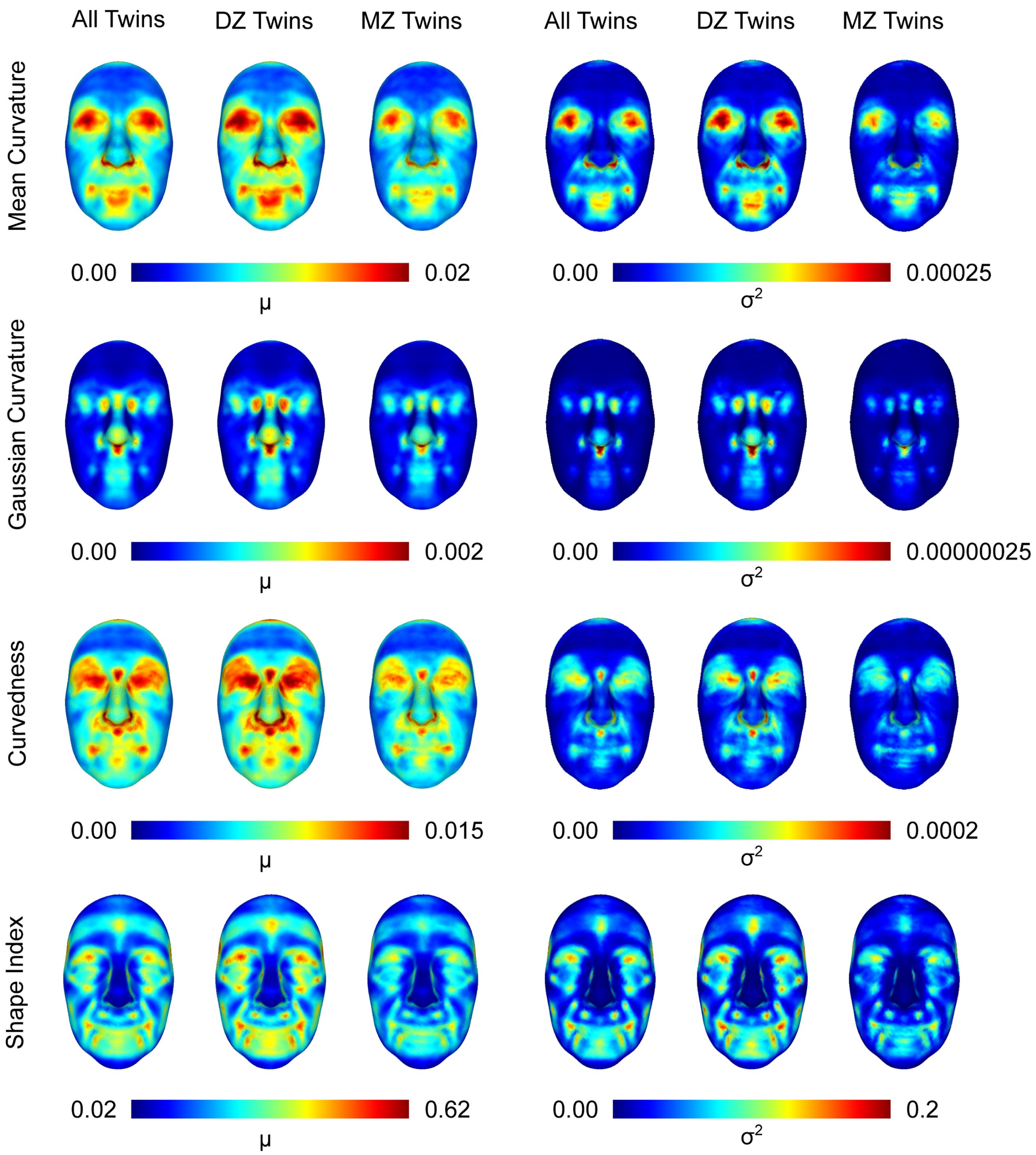


**Figure 5. Mean and variance maps of absolute curvature differences between twins.** For each pair of twins, the absolute difference in curvature values was computed on all facial points. The maps show the mean and variance of the differences for all twins, as well as for MZ and DZ subsets.

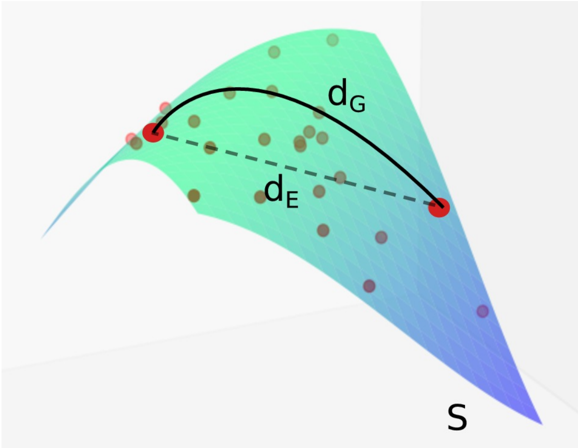


**Figure 6. Illustration example of Geodesic and Euclidean distance metrics.** The distance between two points that lie on a surface $\mathscr{S} \in \mathbb{R}^3$ may be either the length $d_E$ of the straight path between the two points, or the length $d_G$ of the shorted curved path between the same points, under the constraint that movement is only allowed on the surface $\mathscr{S}$.

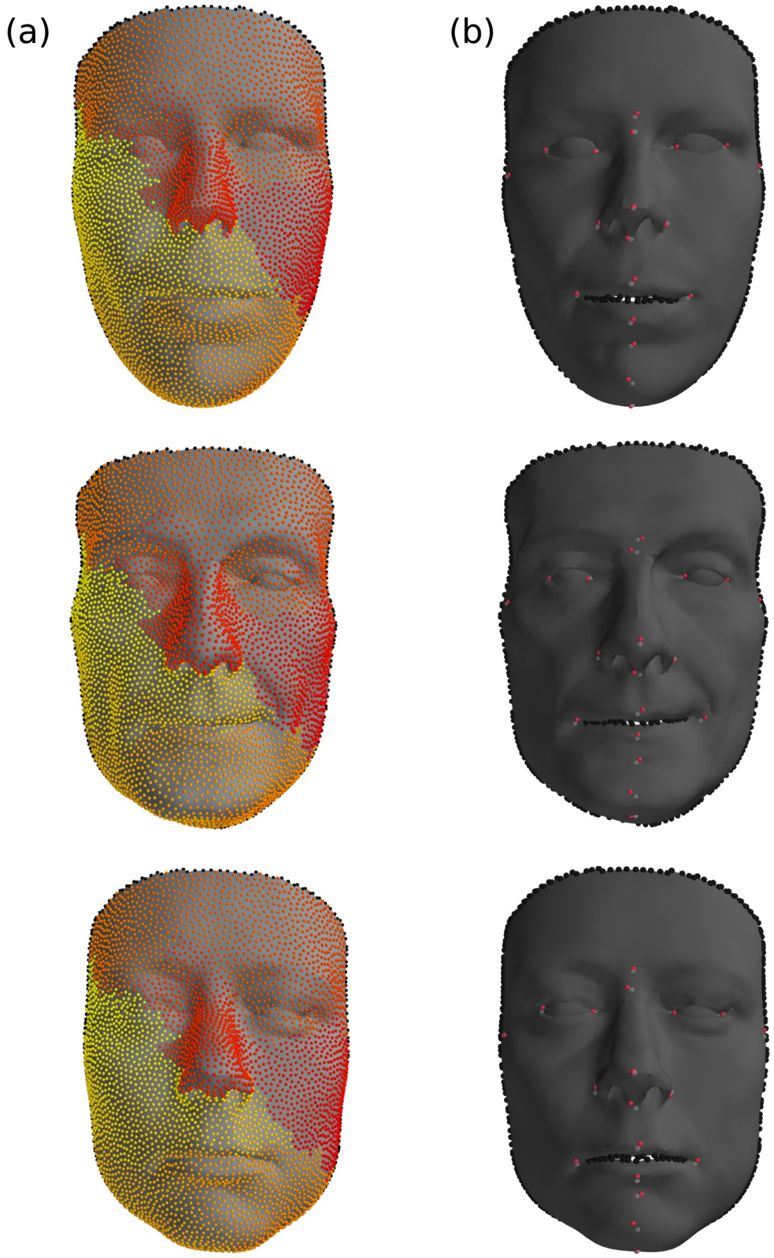


**Figure 7. Extracted landmark sets on validation faces using GESSA.** (A) 4,096 corresponding facial points were computed using our dense automated landmarking method. Results for three validation faces are shown here. Corresponding points are colored consistently among the different faces. Preselected Groundtruth landmarks (GTLs) are shown in white. (B) Red points represent the nearest GESSA generated landmarks (GESLs) to the GTLs for the same example faces. Dense annotation allowed selection of nearest landmarks with distances from GTLs less than 3% of the mean facial width. Importantly, the algorithm was able to consistently place landmarks across different faces.

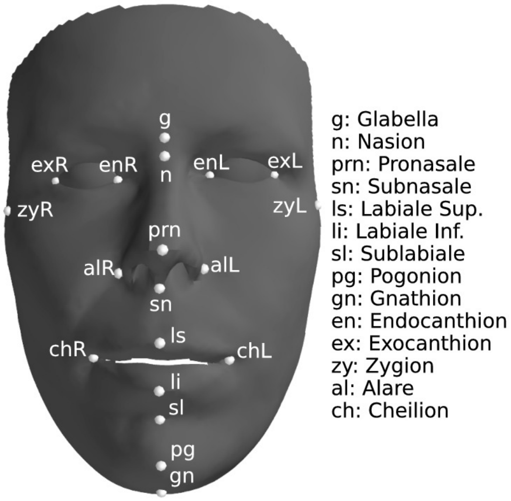


**Figure 8. Morphface Validation Dataset. Example 3D facial surface with groundtruth landmarks.** The 19 groundtruth landmark positions were used during validation of our GESSA landmarking methodology.

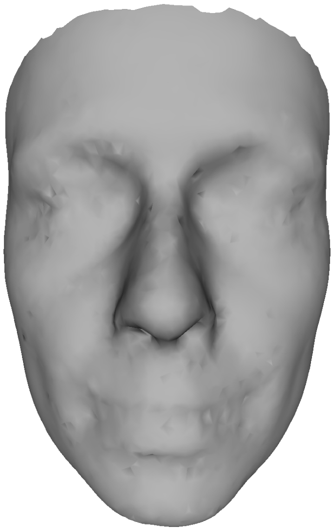

**Figure 9. Morphface Average Facial Surface.** The average surface was constructed by averaging landmark coordinates of the 11 validation faces from the Morphface dataset.

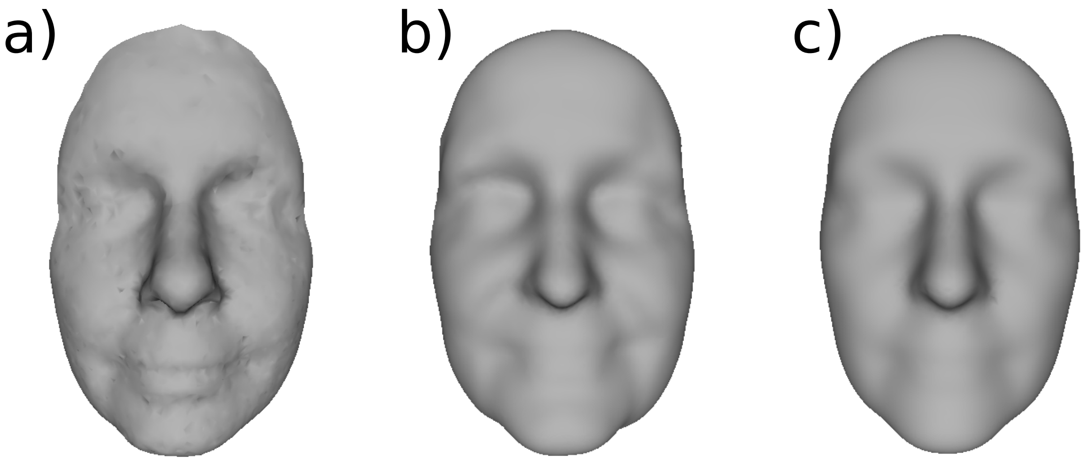


**Figure 10. TwinsUK Average Facial Surfaces.** Three average facial surfaces from the TwinsUK dataset using (a) 10 randomly selected individuals, (b) 200 randomly selected individuals and (c) the complete dataset. Increasing the number of faces results in a smoother average facial surface.

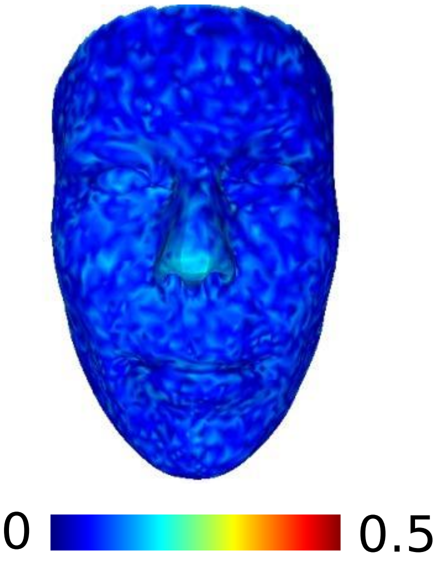


**Figure 11. Estimated Probability Density Function on a Validation Face.** In GESSA, landmarks are considered samples of a random variable defined on the facial surface. By maximizing the sample differential entropy (see Equation (7) in the article), the density function becomes almost constant everywhere. The effect of this optimization is that landmarks are effectively drawn from the uniform distribution.

## 0.2 Supplementary Tables

| | | Landmark Curvature Phenotypes | | | | Composite Curvature Phenotypes | | | |
|---|---|---|---|---|---|---|---|---|---|
| | | MC | GC | CU | SI | MC | GC | CU | SI |
| ACE | $df$ | 5 | 5 | 5 | 5 | 6 | 6 | 6 | 6 |
| | $\chi^2$ | -0.376 | 12.348 | -2.385 | -0.078 | 9.552 | 10.263 | 9.25 | 10.927 |
| | $p$ | 0.573 | 0.442 | 0.576 | 0.603 | 0.29 | 0.273 | 0.29 | 0.251 |
| | $-2\log L$ | 2169.791 | -4186.128 | 1919.943 | 6730.955 | 4850.608 | 4859.602 | 4862.932 | 4868.236 |
| | $AIC$ | 2179.791 | -4176.128 | 1929.943 | 6740.955 | 4858.608 | 4867.602 | 4870.932 | 4876.236 |
| AE | $df$ | 6 | 6 | 6 | 6 | 7 | 7 | 7 | 7 |
| | $\chi^2$ | 0.15 | 12.954 | -1.99 | 0.274 | 10.029 | 11.062 | 9.681 | 11.254 |
| | $p$ | 0.592 | 0.459 | 0.599 | 0.63 | 0.326 | 0.303 | 0.33 | 0.292 |
| | $-2\log L$ | 2170.317 | -4185.521 | 1920.339 | 6731.308 | 4851.084 | 4860.402 | 4863.362 | 4868.563 |
| | $AIC$ | 2178.317 | -4177.521 | 1928.338 | 6739.308 | 4857.084 | 4866.402 | 4869.362 | 4874.563 |
| E | $df$ | 7 | 7 | 7 | 7 | 8 | 8 | 8 | 8 |
| | $\chi^2$ | 70.223 | 66.951 | 56.491 | 41.964 | 99.493 | 91.209 | 86.867 | 83.24 |
| | $p$ | 0.0195 | 0.03 | 0.073 | 0.111 | 0.002 | 0.002 | 0.002 | 0.01 |
| | $-2\log L$ | 2240.391 | -4131.524 | 1978.82 | 6772.997 | 4940.549 | 4940.549 | 4940.549 | 4940.549 |
| | $AIC$ | 2246.391 | -4125.524 | 1984.82 | 6778.997 | 4944.549 | 4944.549 | 4944.549 | 4944.549 |

**Table 1.** Model-Fitting Average Statistics for the Curvature-based Heritability Analyses

| | MC | | GC | | CU | | SI | |
|---|---|---|---|---|---|---|---|---|
| Pearson's $r$ | 6$th$ sPC | MZ 0.720<br>DZ 0.441 | 3$rd$ sPC | MZ 0.709<br>DZ 0.402 | 17$th$ sPC | MZ 0.750<br>DZ 0.411 | 22$th$ sPC | MZ 0.703<br>DZ 0.407 |
| | 43$th$ sPC | MZ 0.714<br>DZ 0.268 | 9$th$ sPC | MZ 0.745<br>DZ 0.450 | 51$th$ sPC | MZ 0.737<br>DZ 0.351 | 27$th$ sPC | MZ 0.704<br>DZ 0.250 |
| | 58$th$ sPC | MZ 0.704<br>DZ 0.363 | 67$th$ sPC | MZ 0.737<br>DZ 0.464 | 11$th$ sPC | MZ 0.727<br>DZ 0.283 | 14$th$ sPC | MZ 0.692<br>DZ 0.365 |
| | 11$th$ sPC | MZ 0.681<br>DZ 0.343 | 15$th$ sPC | MZ 0.694<br>DZ 0.382 | 8$th$ sPC | MZ 0.686<br>DZ 0.428 | 2$nd$ sPC | MZ 0.644<br>DZ 0.445 |
| | 60$th$ sPC | MZ 0.709<br>DZ 0.343 | 4$th$ sPC | MZ 0.695<br>DZ 0.246 | 62$nd$ sPC | MZ 0.697<br>DZ 0.412 | 1$st$ sPC | MZ 0.666<br>DZ 0.338 |

**Table 2.** Top Heritable Composite Curvature Traits - Phenotypic Correlations for MZ and DZ subsets

| | | Linear Distance Phenotypes | Geodesic Distance Phenotypes |
|---|---|---|---|
| ACE | $df$ | 6 | 6 |
| | $\chi^2$ | 13.458 | 15.757 |
| | $p$ | 0.1941 | 0.1678 |
| | $-2\log L$ | 4775.921 | 4834.57 |
| | $AIC$ | 2879.921 | 2938.569 |
| AE | $df$ | 7 | 7 |
| | $\chi^2$ | 16.4 | 17.444 |
| | $p$ | 0.1489 | 0.184 |
| | $-2\log L$ | 4778.863 | 4836.256 |
| | $AIC$ | 2878.862 | 2938.256 |
| E | $df$ | 8 | 8 |
| | $\chi^2$ | 179.8 | 163.724 |
| | $p$ | 0 | 0 |
| | $-2\log L$ | 4942.263 | 4982.537 |
| | $AIC$ | 3042.263 | 3082.536 |

**Table 3.** Model-Fitting Average Statistics for the Distance-based Heritability Analysis

| Phenotype | Ref. | Study | Sample Size | Ethnicity | Related Maps | | |
|---|---|---|---|---|---|---|---|
| NW | [1] | FBH | 229 | Korean | MC Heritability Map | | |
| IED | [1] | FBH | 229 | Korean | 67th GC sPC | 4th GC sPC | 11th CU sPC |
| NP | [2] | TH | 42 | American | 67th GC sPC | 4th GC sPC | 11th CU sPC |
| FW | [3] | TH | 138 | Asian | MC Heritability Map | SI Heritability Map | |
| FW | [4] | FBH | 607 | American | MC Heritability Map | SI Heritability Map | |
| FW | [5] | FBH | 1406 | European | MC Heritability Map | SI Heritability Map | |
| FW | [6] | FBH | 373 | Indian | MC Heritability Map | SI Heritability Map | |
| HC | [7] | FBH | 1042 | European | CU Heritability Map | | |
| HC | [6] | FBH | 373 | Indian | CU Heritability Map | | |
| MRL | [8] | FBH | 363 | European | CU Heritability Map | 60th MC sPC | 14th SI sPC |
| MBL | [8] | FBH | 363 | European | CU Heritability Map | | |
| MR-MB | [8] | FBH | 363 | European | CU Heritability Map | | |
| MR-MB | [9] | TH | 77 | European | CU Heritability Map | | |
| CW | [1] | FBH | 229 | Korean | CU Heritability Map | 15th GC sPC | 8th CU sPC |
| **NW.** Node Width **IED.** Inner Eye Corner Distance **NP.** Nasion Protrusion **FW.** Face Width **HC.** Head Circumference **MRL.** Mandible Ramus Length **MBL.** Mandible Body Length **MR-MB.** Mandible Ramus - Mandible Body Angle **CW.** Chin Width | | | | | | | |
| **FBH.** Family-Based Heritability **TH.** Twin Heritability | | | | | | | |

**Table 4.** Comparison between previously reported heritable phenotypes, heritability maps and composite curvature traits

## 0.3 Supplementary Text

### 0.3.1 Previous Work on Dense Landmarking

Accurate correspondence of landmark points across surfaces is paramount for shape analysis. Until recently, the primary approach to landmark annotation was to manually localize a small number of landmarks on every data object. In the last years, a number of semi-automated and automated landmarking algorithms have appeared in the literature, which attempt to address these issues. In the following we concentrate specifically to methodologies that compute dense landmark correspondences between surfaces, meaning that thousands of landmark points are annotated in each object. This large number of landmarks allows morphological variability to be captured and quantified in a much more granular level, compared to sparse annotations.

Dense landmarking methods can be classified into one of two categories, based on whether annotation is performed in a pairwise fashion - each surface is registered to a common template, with ensemble correspondence extracted implicitly from all one-to-one surface annotations - or a groupwise fashion - all surfaces are considered simultaneously during landmark annotation.

Pairwise methods are more frequently used in the existing literature. The most prominent approach is based on fitting, or warping, a face template or a parametric surface model independently on each of the surfaces in the dataset. 3D Morphable Models[10], Point Distribution Models[11–13] and Nonrigid Iterative Closest Point[14] algorithms are primary examples of this category. Early literature work on these methodologies required an initial manual annotation of a number of landmarks in either a training set of surfaces, or even on the complete dataset. Subsequent extensions attempted to weaken or alleviate this requirement by incorporating the use of additional optimization criterions, such as the minimum description length principle or the maximization of mutual information, see for example[15–18]. Another approach in pairwise landmarking that has risen to attention in recent years entails the use of harmonic or conformal maps which project 3D meshes to a planar domain, thus transforming the 3D correspondence problem to one of 2D image matching[19,20]. An initial annotation of a sparse set of correspondences is still a requirement here, and semi-automated methods have been proposed to achieve that, using for example alignment of mesh vertices through curvature features.

The second class of dense landmarking methodologies attempts to optimize the location of landmark points jointly on all considered surfaces. Although the result is the same as applying one-to-one correspondences between all surfaces in a group and a template or mean surface model, considering all data at once can have certain advantages, such as removing the need for the construction of a template and reducing the bias that can result by the multiple pairwise fits to one surface[21]. Groupwise functional correspondence methods reformulate the landmarking problem as one of finding correspondence between real-valued functional representations of the mesh surfaces, extracted for example using Heat or Wave Kernel Signatures[22–24].

In the work presented by Cates et al.[25], the groupwise correspondence was achieved by optimizing the compactness of the surfaces' distribution, represented as vectors of their landmark coordinates, subject to constraints enforcing uniform distribution of landmarks on individual surfaces. The method was based upon previous work on statistical shape modeling with information-based optimization functions, first presented by Kotcheff and Taylor[26], and subsequent articles by Davies et al.[27,28]. In contrast to the older methods, the algorithm by Cates et al. was not tailored towards the construction of a parametric surface model, but rather to directly solve the landmarking problem. Furthermore, it did not necessitate the use of an anchor surface. Further details and comparisons between there methods can be found in Cates' PhD dissertation entitled 'Shape Modeling and Analysis with Entropy-Based Particle Systems'.

Finally, a number of algorithms, usually coined as groupwise methodologies, adopt an intermediate approach to the correspondence problem. They are based on bottom-up iterative pairwise alignments of similar surfaces, driven by an affinity graph connecting similar surfaces[29,30].

While extensive work has been done in the problem of identifying corresponding landmark points on sets of surfaces, as outlined previously, it is possible to identify some key methodological issues that are still prominent. In pairwise correspondence methods, the construction of a face model, or template, can be a tedious and error-prone procedure. Furthermore, the geometry of the final data objects after correspondence optimization, can be biased towards the mean or template surface. Iterative pairwise methods could address, to a certain extent, such problems, but have not been extensively used so far, probably due the extra computational cost they incur, as well as the difficulty in constructing meaningful affinities between unregistered surfaces. Functional groupwise methodologies, on the other hand, may not suffer from the above issues, but are also associated with specific shortcomings. The most prominent, regarding the problem of point-to-point correspondence, being that the reverse mapping, from corresponding functions, to surface points, is not always easy to construct.

In Cates et al.[25], groupwise correspondence is achieved by manipulating the location of landmark points on the surfaces, such that an objective function comprised of two entropy-based terms is optimized. These terms are related to the uniform distribution of points in each surface and the overall landmark correspondence among surfaces. Due to the fact that landmark annotation under this formulation is equivalent to randomly sampling corresponding points from uniform distributions defined on the surfaces, the problem is also coined with the term ensemble surface sampling. The entropy formulation of the correspondence problem, and the associated optimization procedure, are attractive for a number of reasons. First, the methodology does not necessitate the construction of a template or any manual annotation. Second, the number of computed landmarks can be easily adjusted to the specific application requirements. Third, optimization can be easily tailored to problems of adaptive sampling, as will be discussed in the next section. However, two key problems of the previously presented method could be easily pinpointed. The optimization of the uniform distribution of points on individual surfaces was controlled by a kernel density estimator that did not take into consideration the structure of the surfaces. As a result, the method does not provide an optimal uniform distribution of landmarks and can not deal with highly curved surfaces. In addition, the gradient-descent optimization algorithm also ignored surface constraints. Point updates had to be recast on the surface after each iteration, which further impairs efficiency and performance.

In this work, we extend the methodology of Cates et al.[25] and present our Geodesic Ensemble Surface Sampling Algorithm (GESSA) for the automated identification of landmarks across sets of similar polyhedral surfaces. We propose a suitable estimator for the probability density function of a variable defined on a manifold or polyhedral surface, and employ it in the construction of the objective function. Furthermore, a gradient descent algorithm is constructed, which enables the optimization

to be performed directly on the surfaces. incorporating these two key features inside the existing framework, we are able to to deal with highly curved surfaces and improve upon computational space requirements.

### 0.3.2 Sparse Principal Component Analysis

Different sparse PCA methods have been presented in the literature[31–33]. Here use Penalized Matrix Decomposition (PMD), as proposed in[32], which has been shown to be similar but more computationally efficient than the SCoTLASS sPCA formulation[32,33].

Without loss of generality, let $P$ be a column-wise zero-mean $N \times M$ data matrix. Standard PCA seeks unit loading vectors $v_k$ so that linear transformations - principal components - $Pv_k$ have successively maximum variance. The first PC loading vector is thus computed as

$$v_1 = \arg\max_{v} v^T P^T P v, \ s.t. \ v^T v = 1 \tag{1}$$

Consequent loading vectors can be computed by repeating the same process on the deflated data matrices. Given $P_k$ and $v_k$, the deflated data matrix $P_{k+1} = P_k - P_k v_k v_k^T$, with $P_1 = P$.

The SCoTLASS procedure for sPCA modifies the optimization problem (1) with an additional $L_1$ regularization constraint on the loading vectors: $\|v\|_1 \leq t$, for some tuning parameter $t$. It has been shown that for the first PC, SCoTLASS is equivalent to the following penalized matrix decomposition problem[32]:

$$v_1 = \arg\max_{u,v} u^T P v, \ s.t. \ \|v\|_1 \leq s_p, \ \|v\|_2^2 \leq 1, \ \|u\|_2^2 \leq 1, \tag{2}$$

where $s_p$ is the sparsity parameter, with lower values leading to sparser loading vectors $v$. The above problem is biconvex and can be optimized by iteratively alternating between maximization with respect to $u$ and $v$. SCoTLASS imposes orthogonality constraints between subsequent loading vectors, which though makes optimization very difficult. PMD does not utilize such constraints. Consequent components in PMD are again computed by applying the same procedure on the deflated data matrices.

We notice here that sPCA does not guarantee uncorrelated principal components. We have opted to use sPCA since a main objective in our decomposition analysis was to construct composite traits corresponding to spatially coherent facial areas, which could not be achieved through standard PCA. This coherency was imposed by controlling the sparsity parameter $s_p$. Tuning the parameter is commonly performed through cross validation, by selecting the value that leads to minimum average CV reconstruction error of the data[34]. This process though could have led to spatially extended loading vectors for which biological interpretation would be difficult. Furthermore, the parameter would need tuning for each principal component independently, which would be computationally expensive.

Since our primary objective was to estimate the heritability of principal components, we evaluated the effect of sparsity by comparing heritability estimates (see below for detailed description) of a fixed number of PCs derived from different $s_p$ values. In detail, we computed 100 sPCs for each curvature descriptor and 7 different parameter values. Heritability estimates for all components were subsequently computed. We noticed that the sorted heritability estimates show similar behavior across curvature descriptors for all $s_p$ values tested.

Based on the fact that heritability estimation would not be significantly affected by the particular value of $s_p$, we selected constant values for each descriptor after visual inspection of sPC loading maps - constructed by mapping the sPCs' loadings on the facial surface - with the criterion of which parameter yielded sPCs more suitable for further biological interpretation. In particular, the parameter was set to 7.5 for GC, 12.5 for SI and 15 for MC and CU.

We retained for further analysis all 100 sPCs for each curvature descriptor. Each set of sPCs was able to cumulatively explain respectively 92.72%, 89.41%, 90.11% and 88.2% of the MC, GC, CU and SI phenotypic curvature variance.

### 0.3.3 Structural Equation Modelling

In this work, we estimated heritability as the proportion of phenotypic variance explained by genetic factors. Since the genetic and environmental variables are unobserved (latent), their effects are inferred from twin resemblance using Structural Equation Modelling (SEM). SEM encompasses a broad family of statistical modeling techniques and can be viewed as a combination of path and latent factor analysis[35]. SEM is also widely referred to as covariance structure modeling, since a SE model implies a structure for the covariances between observed variables.

In heritability studies, observed phenotypic variation can be partitioned into variance components from the following latent factors: additive (A) genetic, dominant (D) genetic, common (C) environmental and unique (E) environmental, with the latter component also including measurement error. A path diagram of a SEM including all of the above latent factors can be seen in Figure 12. The structure of covariance is implied by latent factor correlations between twin pairs. Additive and dominant genetic effects are correlated 1 between MZ pairs, while only 0.5 and 0.25 respectively between DZ pairs. Common environmental effects have correlation 1 for both types of twins while unique environmental factors are uncorrelated.

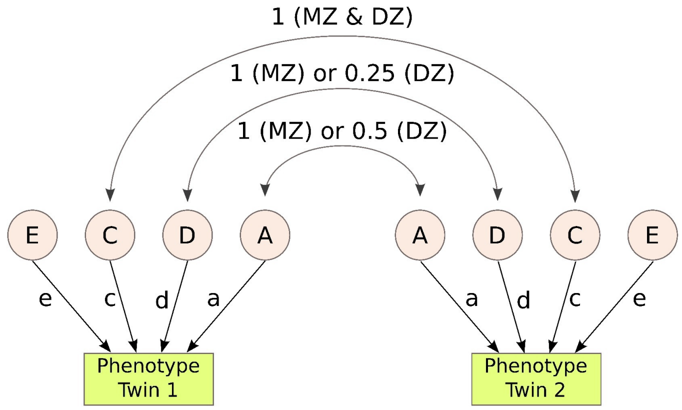


**Figure 12. Path Diagram of the ACDE Structural Equation Model.** Latent factors $A$, $D$, $C$ and $E$ correspond to additive and dominant genetic, common and unique environmental effects respectively. Double arrows represent the latent factor correlations between pairs of twins. Additive and dominant genetic effects are correlated 1 between MZ pairs, 0.5 and 0.25 between DZ pairs. Common environmental effects have correlation 1 for both types of twins while unique environmental factors are uncorrelated. $a$, $d$, $c$ and $e$ are regression path coefficients of the respective latent factors. Heritability in a ACDE SEM model is given by $h^2_{ACDE} = \frac{a^2+d^2}{a^2+d^2+c^2+e^2}$.

For twin studies in particular, the model ADCE is over-specified and cannot be estimated using twin data alone[36]. Submodels that can be examined are ACE, ADE, AE, CE and E. Models composed of the components DE are not considered biologically plausible. Here we considered ACE, AE and E models. As such, below we focus and describe in detail the definition of the ACE SE model.

A univariate ACE model can be expressed as

$$P = aA + cC + eE, \tag{3}$$

where for simplicity and without loss of information, $P$ is an observed zero-centered, continuous phenotypic variable and $A$, $C$, $E$ are unobserved latent factors with fixed unit variances and covariances that depend on the type of twin. Finally, $a$, $c$, $e$ are regression coefficients expressing the effects of the latent variables in the phenotype. Now Let $P_{MZ}$ be a $N_{MZ} \times 2$ matrix of phenotypic observations with each row coming from one pair of MZ twins and $N_{MZ}$ the number of MZ paired observations. Respectively $P_{DZ}$ denotes a $N_{DZ} \times 2$ matrix of phenotypic observations with each row coming from one pair of DZ twins. Furthermore let

$$L = \begin{bmatrix} a & 0 \\ c & 0 \\ e & 0 \\ 0 & a \\ 0 & c \\ 0 & e \end{bmatrix}, \tag{4}$$

be the matrix of regression coefficients and finally $\Lambda_{MZ}$, $\Lambda_{DZ}$ be $N_{MZ} \times 6$ and $N_{DZ} \times 6$ matrices of unobserved A, C, E factors for MZ and DZ twin subsets respectively.

Expressing the ACE model for our observations, we have

$$P_{MZ} = \Lambda_{MZ} L, \; P_{DZ} = \Lambda_{DZ} L. \tag{5}$$

The expected phenotypic covariances from the ACE model are:

$$\begin{aligned} \Sigma_{MZ} &= E[\Lambda_{MZ}^T \Lambda_{MZ}] = L^T \Psi_{MZ} L \\ \Sigma_{DZ} &= E[\Lambda_{DZ}^T \Lambda_{DZ}] = L^T \Psi_{DZ} L, \end{aligned} \tag{6}$$

where the correlation matrices $\Psi_{MZ}$, $\Psi_{DZ}$ of the latent factors are derived from the SEM path diagram. In particular, $\Psi_{MZ}$ has two off diagonal elements equal to 1, corresponding to $corr(A_1, A_2)$ and $corr(C_1, C_2)$, while $\Psi_{DZ}$ has $corr(A_1, A_2) = 0.5$ and $corr(C_1, C_2) = 1$ (see Figure 12).

The structured covariance matrices as modelled by SEM can be now easily computed to be

$$\Sigma_{MZ} = \begin{bmatrix} a^2+c^2+e^2 & a^2+c^2 \\ a^2+c^2 & a^2+c^2+e^2 \end{bmatrix}, \tag{7}$$

$$\Sigma_{DZ} = \begin{bmatrix} a^2+c^2+e^2 & 0.5a^2+c^2 \\ 0.5a^2+c^2 & a^2+c^2+e^2 \end{bmatrix}, \tag{8}$$

Maximum Likelihood is used to estimate the regression coefficients $a$, $c$, $e$. Let $S_{MZ}$ and $S_{DZ}$ be the observed sample covariances. Assuming that the phenotypic response is normally distributed, the probabilities of observing $S_{MZ}$ and $S_{DZ}$ given estimates $\hat{\Sigma}_{MZ}$ and $\hat{\Sigma}_{DZ}$ follow the Wishart distribution with $N_{MZ}$ and $N_{DZ}$ degrees of freedom respectively. The log-likelihood functions can be written as follows, after the omission of constant terms[37]:

$$\begin{aligned} -2LL_{MZ} &\approx N_{MZ}\left[\ln|\hat{\Sigma}_{MZ}| + tr(\hat{\Sigma}_{MZ}^{-1}S_{MZ})\right] \\ -2LL_{DZ} &\approx N_{DZ}\left[\ln|\hat{\Sigma}_{DZ}| + tr(\hat{\Sigma}_{DZ}^{-1}S_{DZ})\right]. \end{aligned} \tag{9}$$

Estimates $\hat{a}, \hat{c}, \hat{e}$ are obtained by maximizing the combined likelihood function $-2(LL_{MZ} + LL_{DZ})$. Model fit can be assessed using a log-likelihood ratio test between the structured model and a fitted saturated model where no structure is imposed on the covariances. The ratio statistic is distributed approximately as a chi-squared distribution with degrees of freedom equal the difference in $df$ between the structured and the saturated model.

At this point we can also define the heritability estimate $h^2_{ACE}$ from the ACE model as

$$h^2_{ACE} = \frac{\hat{a}^2}{\hat{a}^2+\hat{c}^2+\hat{e}^2}. \tag{10}$$

An important aspect of SEM in twin studies is that the significance of individual variance components can be assessed by dropping parameters sequentially from nested models; here ACE→AE→E. In choosing between models, variance components are excluded in the selection process if there is no significant deterioration in model fit, assessed commonly by the Akaike Information Criterion (AIC)[38], after the component is dropped. The E component represents random error and is always retained[36]. Heritability is estimated from the AE model as

$$h^2_{AE} = \frac{\hat{a}^2}{\hat{a}^2+\hat{e}^2}. \tag{11}$$

In this study, we estimated heritability for all 4,096 curvature traits independently, as well as for the top 100 variance-explaining sPCs (composite traits), for each curvature descriptor, using SEM. We assessed the significance of individual variance components by dropping parameters sequentially from the set of nested models ACE, AE and E, fitted using the OpenMx software[39,40]. Age was included in the models as a covariate.

# References


1. Kim, H.-J. *et al.* Heritabilities of facial measurements and their latent factors in Korean families. *Genomics & informatics* **11**, 83–92 (2013).
2. Weinberg, S. M., Parsons, T. E., Marazita, M. L. & Maher, B. S. Heritability of face shape in twins: a preliminary study using 3D stereophotogrammetry and geometric morphometrics. *Dentistry 3000* **1** (2013).
3. Baydaş, B., Erdem, A., Yavuz, I. & Ceylan, I. Heritability of facial proportions and soft-tissue profile characteristics in Turkish Anatolian siblings. *American journal of orthodontics and dentofacial orthopedics* **131**, 504–509 (2007).
4. Sherwood, R. J. *et al.* Quantitative genetics of modern human cranial variation. *Journal of human evolution* **54**, 909–914 (2008).
5. Ermakov, S., Kobyliansky, E. & Livshits, G. Quantitative genetic study of head size related phenotypes in ethnically homogeneous chuvasha pedigrees. *Annals of human biology* **32**, 585–598 (2005).
6. Karmakar, B., Ermakov, S., Yakovenko, K. & Kobyliansky, E. Genetic determination of head-size-related anthropometric traits in an ethnically homogeneous sample of 373 Indian pedigrees of west Bengal. *Human biology* **79**, 501–514 (2007).
7. Ermakov, S., Rosenbaum, M. G., Malkin, I. & Livshits, G. Family-based study of association between ENPP1 genetic variants and craniofacial morphology. *Annals of human biology* **37**, 754–766 (2010).

**8.** Johannsdottir, B., Thorarinsson, F., Thordarson, A. & Magnusson, T. E. Heritability of craniofacial characteristics between parents and offspring estimated from lateral cephalograms. *American Journal of Orthodontics and Dentofacial Orthopedics* **127**, 200–207 (2005).

**9.** Carels, C. *et al.* A quantitative genetic study of cephalometric variables in twins. *Clinical orthodontics and research* **4**, 130–140 (2001).

**10.** Blanz, V. & Vetter, T. A morphable model for the synthesis of 3D faces. In *Proceedings of the 26th annual conference on Computer graphics and interactive techniques*, 187–194 (ACM Press/Addison-Wesley Publishing Co., 1999).

**11.** Claes, P., Walters, M. & Clement, J. Improved facial outcome assessment using a 3D anthropometric mask. *International journal of oral and maxillofacial surgery* **41**, 324–330 (2012).

**12.** Ghosh, D., Sharf, A. & Amenta, N. Feature-driven deformation for dense correspondence. In *SPIE Medical Imaging*, 726136–726136 (International Society for Optics and Photonics, 2009).

**13.** Hutton, T. J. *Dense surface models of the human face*. Ph.D. thesis, Citeseer (2004).

**14.** Amberg, B., Romdhani, S. & Vetter, T. Optimal step nonrigid ICP algorithms for surface registration. In *2007 IEEE Conference on Computer Vision and Pattern Recognition*, 1–8 (IEEE, 2007).

**15.** Thodberg, H. H. Minimum description length shape and appearance models. In *Information Processing in Medical Imaging*, 51–62 (Springer, 2003).

**16.** Rueckert, D. *et al.* Nonrigid registration using free-form deformations: application to breast MR images. *IEEE transactions on medical imaging* **18**, 712–721 (1999).

**17.** Guo, J., Mei, X. & Tang, K. Automatic landmark annotation and dense correspondence registration for 3D human facial images. *BMC bioinformatics* **14**, 232 (2013).

**18.** Kakadiaris, I. A. *et al.* Three-dimensional face recognition in the presence of facial expressions: An annotated deformable model approach. *IEEE Transactions on Pattern Analysis and Machine Intelligence* **29**, 640–649 (2007).

**19.** Wang, S., Wang, Y., Jin, M., Gu, X. D. & Samaras, D. Conformal geometry and its applications on 3D shape matching, recognition, and stitching. *IEEE Transactions on Pattern Analysis and Machine Intelligence* **29**, 1209–1220 (2007).

**20.** Wang, Y. *et al.* High resolution tracking of non-rigid motion of densely sampled 3D data using harmonic maps. *International Journal of Computer Vision* **76**, 283–300 (2008).

**21.** Van Kaick, O., Zhang, H., Hamarneh, G. & Cohen-Or, D. A survey on shape correspondence. In *Computer Graphics Forum*, vol. 30, 1681–1707 (Wiley Online Library, 2011).

**22.** Ovsjanikov, M., Ben-Chen, M., Solomon, J., Butscher, A. & Guibas, L. Functional maps: A flexible representation of maps between shapes. *ACM Transactions on Graphics (TOG)* **31**, 30 (2012).

**23.** Zhang, C., Smith, W. A., Dessein, A., Pears, N. & Dai, H. Functional faces: Groupwise dense correspondence using functional maps. In *Proceedings of the IEEE Conference on Computer Vision and Pattern Recognition*, 5033–5041 (2016).

**24.** Bronstein, A. M., Bronstein, M. M. & Kimmel, R. *Numerical geometry of non-rigid shapes* (Springer Science & Business Media, 2008).

**25.** Cates, J., Fletcher, T. P., Styner, M., Shenton, M. & Whitaker, R. Shape modeling and analysis with entropy-based particle systems. In *Information Processing in Medical Imaging*, 333–345 (Springer, 2007).

**26.** Kotcheff, A. C. & Taylor, C. J. Automatic construction of eigenshape models by direct optimization. *Medical Image Analysis* **2**, 303–314 (1998).

**27.** Davies, R. H., Twining, C. J., Cootes, T. F., Waterton, J. C. & Taylor, C. J. A minimum description length approach to statistical shape modeling. *Medical Imaging, IEEE Transactions on* **21**, 525–537 (2002).

**28.** Davies, R. H., Twining, C. J., Cootes, T. F., Waterton, J. C. & Taylor, C. J. 3d statistical shape models using direct optimisation of description length. In *European conference on computer vision*, 3–20 (Springer, 2002).

**29.** Tang, Z., Jiang, D. & Fan, Y. Image registration based on dynamic directed graphs with groupwise image similarity. In *Biomedical Imaging (ISBI), 2013 IEEE 10th International Symposium on*, 492–495 (IEEE, 2013).

**30.** Wang, Q., Chen, L., Yap, P.-T., Wu, G. & Shen, D. Groupwise registration based on hierarchical image clustering and atlas synthesis. *Human brain mapping* **31**, 1128–1140 (2010).

**31.** Zou, H., Hastie, T. & Tibshirani, R. Sparse principal component analysis. *Journal of computational and graphical statistics* **15**, 265–286 (2006).

**32.** Witten, D. M., Tibshirani, R. & Hastie, T. A penalized matrix decomposition, with applications to sparse principal components and canonical correlation analysis. *Biostatistics* kxp008 (2009).

**33.** Jolliffe, I. T., Trendafilov, N. T. & Uddin, M. A modified principal component technique based on the lasso. *Journal of computational and Graphical Statistics* **12**, 531–547 (2003).

**34.** Witten, D., Tibshirani, R., Gross, S., Narasimhan, B. & Witten, M. D. Package PMA. *Genetics and Molecular Biology* **8**, 28 (2013).

**35.** Hox, J. J. An introduction to structural equation modeling. *Family Science Review* **11**, 354–373.

**36.** Rijsdijk, F. V. & Sham, P. C. Analytic approaches to twin data using structural equation models. *Briefings in bioinformatics* **3**, 119–133 (2002).

**37.** Martin, N. G. & Eaves, L. J. The genetical analysis of covariance structure. *Heredity* **38**, 79 (1977).

**38.** Akaike, H. A new look at the statistical model identification. *Automatic Control, IEEE Transactions on* **19**, 716–723 (1974).

**39.** Boker, S. *et al.* OpenMx: An open source extended structural equation modeling framework. *Psychometrika* **76**, 306–317 (2011).

**40.** Openmx - advanced structural equation modeling [internet]. Available from: `http://openmx.psyc.virginia.edu/`. [Access date].
